# Global offshore wind turbine dataset

**DOI:** 10.1038/s41597-021-00982-z

**Published:** 2021-07-27

**Authors:** Ting Zhang, Bo Tian, Dhritiraj Sengupta, Lei Zhang, Yali Si

**Affiliations:** 1 State Key Laboratory of Estuarine and Coastal Research, East China Normal University, 200062 Shanghai, China; 2 Department of Traffic Information and Control Engineering, Tongji University, 201804 Shanghai, China; 3 Institute of Environmental Sciences, Leiden University, 2333 CC Leiden, Netherlands

**Keywords:** Environmental impact, Sustainability, Energy management, Energy access, Energy policy

## Abstract

Offshore wind farms are widely adopted by coastal countries to obtain clean and green energy; their environmental impact has gained an increasing amount of attention. Although offshore wind farm datasets are commercially available via energy industries, records of the exact spatial distribution of individual wind turbines and their construction trajectories are rather incomplete, especially at the global level. Here, we construct a global remote sensing-based offshore wind turbine (OWT) database derived from Sentinel-1 synthetic aperture radar (SAR) time-series images from 2015 to 2019. We developed a percentile-based yearly SAR image collection reduction and autoadaptive threshold algorithm in the Google Earth Engine platform to identify the spatiotemporal distribution of global OWTs. By 2019, 6,924 wind turbines were constructed in 14 coastal nations. An algorithm performance analysis and validation were performed, and the extraction accuracies exceeded 99% using an independent validation dataset. This dataset could further our understanding of the environmental impact of OWTs and support effective marine spatial planning for sustainable development.

## Abstract (summary)

**Table:** 

Measurement(s)	offshore wind turbine
Technology Type(s)	satellite imaging • digital curation
Factor Type(s)	temporal interval • spatial extent
Sample Characteristic - Environment	wind farm • atmospheric wind
Sample Characteristic - Location	global

Machine-accessible metadata file describing the reported data: 10.6084/m9.figshare.14865690

## Background & Summary

Offshore wind farms, which comprise a cluster, or array, of wind turbines, is widely accepted as renewable sources of energy and effective ways to reduce greenhouse gas emissions and promote a net-zero carbon economy. In recent years, 14 countries around the world have installed offshore wind farms on their coastal frontier. To date, although offshore wind farms cover only approximately 8% of the global renewable energy market and approximately 3.5% of the global installed capacity, these numbers will increase substantially in the next few years^1,2^. Using the clean energy generated by offshore wind farms can help to achieve Intergovernmental Panel on Climate Change (IPCC) targets and meet the Sustainable Development Goals (SDGs) by regulating emissions and promoting developments in the renewable energy sector (Goal 13), hence ensuring access to affordable, reliable, sustainable and modern energy for all (Goal 7).

Nevertheless, the potential environmental impacts of offshore wind farms, which are currently still under debate^3,4^, should be further investigated. To reduce the costs of construction and maintenance, most OWTs are located in close proximity to the coast. This area is very sensitive due to its influence on marine mammals, phytoplankton^5^, birds^6,7^, fish^8^, and invertebrates^9,10^, as well as the landscapes of local communities^11^. The spatial distribution and construction trajectory of wind turbines are prerequisites for environmental impact assessments to guide OWT spatial planning. This assessment directly involves the interest of developers, operators, and owners to balance income from renewable energy with ecological protection, thereby ensuring that OWTs are truly ecologically friendly and sustainable to meet the growing demand of energy.

To the best of our knowledge, there are 35 regional, national or international renewable energy databases that include OWT data^12–16^, including 11 international databases and 24 regional/national databases. The international offshore wind farm datasets, such as the 4 C Offshore Wind Database^17^ or The Wind Power^18^, contain project details for more offshore wind projects than other databases but are partly open and need to be paid when collecting high resolution information about these wind farm locations. Although open international offshore wind farm datasets, such as the global datasets of wind and solar farms (GBWSFs) built by Dunnett *et al*^14^., can be freely accessed, there are obvious omissions of turbine numbers and recording errors of wind turbine locations. For example, this dataset omits 70% (of the 50 wind farms, 35 are missing) offshore wind farm information (i.e., the wind turbine number and specific spatial location information) when compared with the 4 C Offshore Wind Database^17^, United States Geological Survey (USGS) Wind Turbine Dataset (USWTD)^15^, United Kingdom Renewable Energy Planning Database (UK REPD)^16^, European Marine Observation and Data Network (EMODnet) wind farm database^19^ and Open Power System Data (OPSD) renewable power plant database^13^ (refer to details in Online-only Table 1). Among these regional/national databases, the USWTD and OPSD provide the exact OWTs location. Although they do not have global coverage and updates of the latest installations, part of these data, such as the USA (USWTD) and Germany (OPSD) wind farms data, have been validated and can be referenced. Other databases, such as EMODnet, provide the number of turbines and spatial boundaries or the centroids of wind farms but lack information on their precise locations, while the UKREPD also suffers from inaccuracy of location, and only has an approximate centroid for each offshore wind farm. Therefore, to date, no global OWT dataset with accurate geographic turbine location information is available in the public domain.

Satellite imagery is an important source of information for the identification of OWTs. However, widely utilized passive optical images (i.e., Landsat 30-m resolution images) are often affected by clouds and mist over coastal zones, which makes it difficult to map wind turbines^20^. In contrast, SAR data from the Sentinel-1A/B satellite, which was launched by the European Space Agency in 2014, can collect information regardless of cloud cover, day or night and can be used to identify OWT objects, in which the presence of dihedral structures results in a drastic increase in backscattering^21^.

**Fig. 1:**
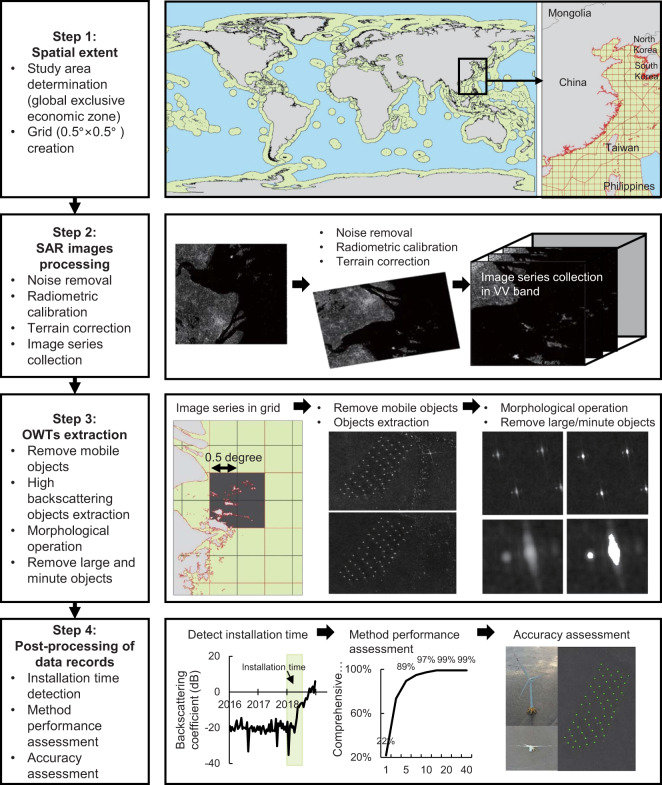
Flow diagram showing the data acquisition and processing procedures in generating the global offshore wind turbine (OWT) dataset.

In this study, we build a global OWT dataset by applying a percentile-based yearly SAR image collection reduction and autoadaptive threshold algorithm on the Google Earth Engine (GEE) platform using more than 737,100 Sentinel-1 SAR images. A method performance analysis, validation assessment and accuracy analysis were performed using Google high-resolution imagery, multisource optical and radar satellite image data (i.e., Landsat 8-OLI, Sentinel 2-MSI, Sentinel 1 data), ground unmanned real-time kinematic (RTK) drone investigation and other datasets (i.e., 4 C Offshore, USWTD, UK REPD, OPSD, EMODnet). Figure 1 depicts the data acquisition and processing steps using a flow diagram. Compared to the offshore wind farm dataset extracted or validated by aerial imagery, the wind turbine number obtained by our global OWF dataset will not be underestimated since available Sentinel 1 data do not lag actual installations by several months. Therefore, this dataset can also be used to analyse regional variations in OWFs, prioritize OWF planning, and assess their potential environmental impacts. The global OWF dataset will be updated annually and is currently free to download via Figshare^22^.

## Methods

The global OWT dataset was developed by using geospatial technology and advanced mathematical operations on the GEE platform using earth observation Sentinel 1 SAR time-series imagery. These operations were performed to map the spatial distribution of individual OWT in the global coastal zone.

### Spatial extent

The spatial extent of OWTs covers the global offshore area in each exclusive economic zone (EEZ)^23^. The EEZ database provides the maritime boundary prescribed by the 1982 United Nations Convention on the Law of the Sea over which a sovereign state has special rights regarding the exploration and use of marine resources. Based on this database, the extraction of OWTs was organized into 0.5° × 0.5° vector grids for the global coast. The main reason for this step was to reduce the computational memory of remote servers on the GEE platform as well as to select a systematic geographic extent for this study.

### SAR image processing

SAR images were collected and processed on the GEE platform. Imagery in the GEE ‘COPERNICUS/S1_GRD’ Sentinel-1 image collection consists of Level-1 Ground Range Detected (GRD) scenes, which process the backscatter coefficient (σ°) in decibels (dB). Each scene in GEE was preprocessed with the Sentinel-1 Toolbox using the following steps: (1) application of an orbit file that updates the orbit metadata with a restituted orbit file; (2) removal of low-intensity GRD border noise and invalid data on scene edges; (3) thermal noise removal, which eliminates additive noise in subswaths to help reduce discontinuities between subswaths for scenes in multiswath acquisition modes; (4) radiometric calibration, which computes the backscatter intensity using the sensor calibration parameters in the GRD metadata; and (5) terrain correction using Shuttle Radar Topography Mission (SRTM) or Advanced Spaceborne Thermal Emission and Reflection Radiometer (ASTER) digital elevation model (DEM) products. This procedure basically converts data from ground range geometry. The concluding terrain-corrected figures are transformed to decibels through log scaling (10*log10(x)).

**Fig. 2:**
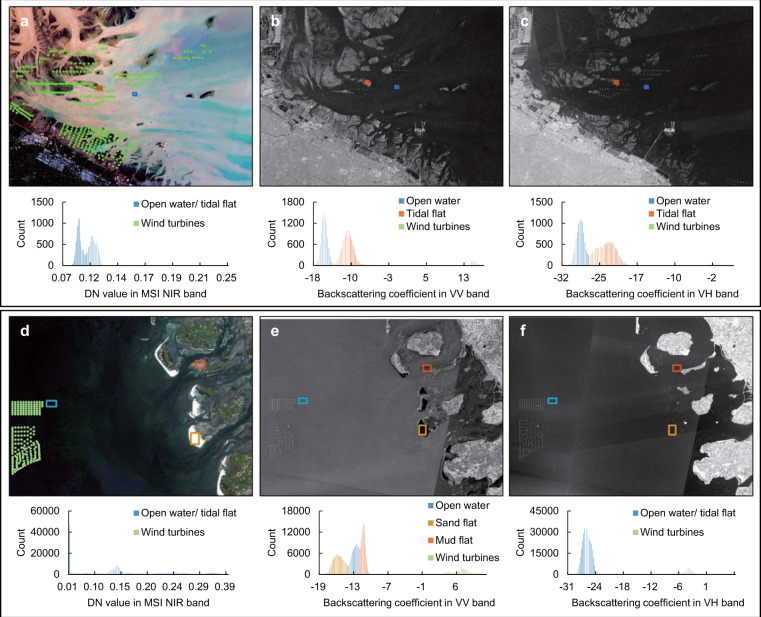
Extractability analysis of OWTs in the East China Sea (top panel) and North Sea (bottom panel). Each panel consists of a Sentinel-2 MSI true-colour image (**a** and **d**) and a Sentinel-1 image in vertical-vertical (VV, **b** and **e**) and vertical-horizontal (VH, **c** and **f**) polarization modes. Individual histograms show the digital number (DN) and backscatter values for the regions of interest highlighted in green, blue and red, which correspond to wind turbines, open water and tidal mud/sand flats, respectively.

In this study, Sentinel-1 imagery from interferometric wide (IW) swath mode and in vertical-vertical (VV) polarization is selected for the analyses. This configuration was selected because it is more effective in detecting offshore emissions, as shown in Fig. 2, than other configurations. We selected three regions of interest for three types of objects in the offshore areas of the East China Sea and the North Sea, including tidal flats, open water and OWTs. The histogram distribution of the digital number (DN) values in the near-infrared band of the Sentinel-2 MultiSpectral Instrument (MSI) and the backscattering coefficients in the Sentinel-1 VV and vertical-horizontal (VH) polarization bands of these regions are compared. The results showed that the backscattering coefficients of wind turbines in the Sentinel 1 VV band have higher separability when distinguishing them from open water and tidal flats. From Fig. 2, it is obvious that if the maximum backscatter coefficient is less than 0 dB in a particular grid, then this grid does not contain a wind farm. Therefore, we can directly exclude some grids from the analysis according to the following criterion in Eq. (1):

1 $$\begin{array}{l}{\rm{Grid}}\left(retain\;or\;not\right)=\left\{\begin{array}{ll}{\rm{exclude}} & if\;B{C}_{max}\le 0\;(dB)\\ include & otherwise\end{array}\right.\\ \end{array}$$

### OWTs extraction

**Fig. 3:**
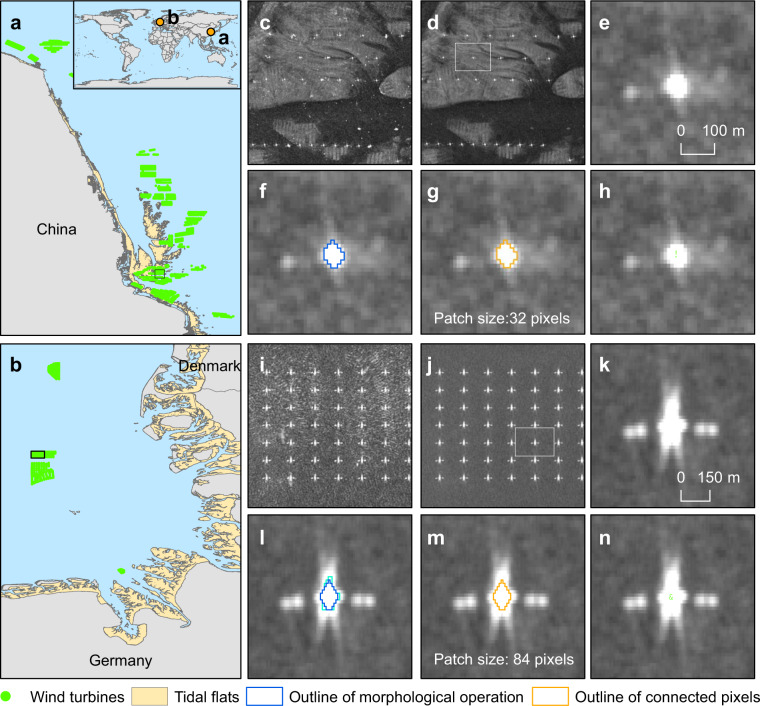
Illustration of the steps used in OWTs extraction. (**a**) The East China Sea; (**b**) The North Sea. (**c**) and I: Original SAR images, (**d** and **j**) remove mobile objects, (**e** and **k**) extract high-backscatter objects, (**f** and **l**) apply morphological processing, (**g** and **m**) remove large and very small objects, (**h** and **n**) output the final turbine location.

OWTs extraction was performed systematically by applying five steps: (1) removal of floating or temporarily mobile objects, (2) extraction of high-backscatter objects, (3) morphological operations, (4) removal of large and very small objects, and (5) postprocessing of data records. A detailed explanation of each segment is as follows (Fig. 3):

**1) Removal of floating or temporarily mobile objects**

Taking advantage of the Sentinel-1 time-series data, advanced statistical analysis was applied to the composite images. After preprocessing the Sentinel-1 data and storing them as an ‘ImageCollection’, we filtered them by a date range and spatial boundary to obtain an annual composite of ‘VV’ images for each selected grid. The percentile and mean values of a series are commonly used in statistical measures that we applied to identify floating or temporarily mobile objects based on the frequency of appearance in the image. We then removed floating or temporarily mobile objects, such as ships and vessels, by comparing their mobility with stable objects, such as OWTs. The percentile and interval mean values between 80–100% were applied to the features in the series using the ‘intervalMean()’ reduction method on the GEE platform.

**2) Extraction of high-backscatter objects**

Selection of an optimal threshold value is the most important step in object extraction. However, because of the variability in global ocean water on the SAR backscatter coefficient, it is necessary to apply an autoadaptive threshold to different ocean regions. The histogram for a grid without wind turbines generally has one peak in the lower values (water body) and no peak in the higher values (OWTs usually have values greater than 0 dB), which can be reflected from the median of the lowest and highest values. We used a grid-based backscatter filter with an automatic adaptive threshold (*T)* to distinguish high-backscatter objects from the different ocean water backgrounds having low backscatter values. The threshold is defined as the median of the lowest and highest values, termed here the ‘half min-max threshold’. We then obtained a binary image based on the comparison of the backscatter coefficient (Eq. (2)) with *T* (Eq. (3)), and the equation is defined as follows:

2 $${\rm{Binary}}\;{\rm{decision}}=\left\{\begin{array}{c}\begin{array}{cc}1 & if\;BC\ge T\end{array}\\ \begin{array}{cc}0 & otherwise\end{array}\end{array}\right.$$

3 $${\rm{T}}=\frac{B{C}_{max}+B{C}_{min}}{2}$$

where *T* is the dynamic threshold*, BC* is the backscatter coefficient of each pixel in the grid, *BC*_*max*_ is the backscatter maximum, and *BC*_*min*_ denotes the minimum value in the grid.

**3) Morphological operation**

Because the binary images produced by the previous step are distorted by noise and textures, a morphological analysis was employed to enhance the high-backscatter image objects. Morphological processing methods for erosion and dilation can correct these distortions by accounting for the form and structure of the image. Both erosion and dilation processing techniques are a collection of nonlinear operations related to the shape or morphology of features in an image. The value of the output pixel for dilation is the maximum value of all the pixels in the neighbourhood, which makes objects more visible and fills in small holes in the objects. The value of the output pixel for erosion is the minimum value of all the pixels in the neighbourhood, which removes islands and small objects so that only substantive objects remain.

**4) Removal of large and minute objects**

Knowing the number of pixels in an object can be helpful for masking irrelevant objects of different sizes. An area-size-range filter algorithm (20 < number of pixels < 200) was used to eliminate large and very small objects such as islands, oil platforms and small noise objects. In the GEE platform, the ‘connectedPixelCount()’ method was used to compute the number of pixels in each object.

**5) Post-processing of data records**

**Fig. 4:**
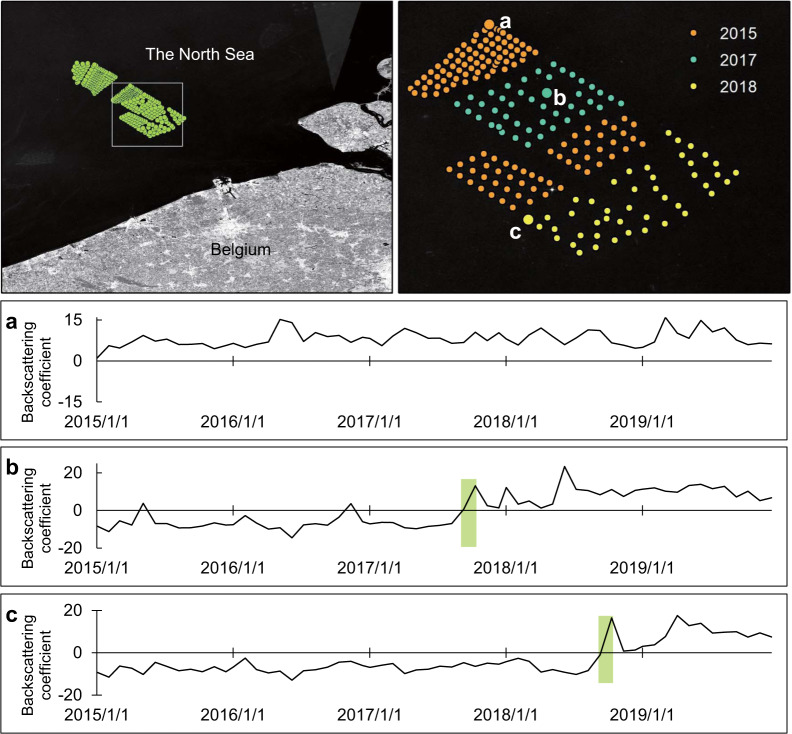
Locations of the OWTs in Belgium (top panel), where a, b and c are the curves of the backscattering coefficient over time. The wind turbines in a appeared before 2015, b was constructed in approximately 2017, and c was built in 2018.

We converted the raster to the vector data type (using the ‘image.reduceToVectors()’ method in the GEE platform) and obtained the latitude and longitude coordinates for the individual wind turbines. As OWTs are constructed, the backscatter coefficient increases rapidly, and hence, the information about the installation dates of the wind turbine foundations can also be extracted from yearly ‘VV’ images. Figure 4 shows an example of the changes in a wind farm constructed in Belgium in different years, and the backscatter coefficient increases when wind turbines are installed. The identification of drastic annual change points was performed by the Mann-Kendall (MK) test^24,25^ over each wind turbine. Individual wind turbines were taken as the central point to make a buffer area of 200 m, which was used to extract the maximum backscatter coefficient as the input parameter of the MK test. This step was performed to avoid the mismatch of the extracted wind turbine central position due to image shifts over time. The MK test is a nonparametric statistical test for which *UF*_*k*_ and *UB*_*k*_ are two important time statistics; here, the statistical sequence *UF*_*k*_ is the result of the backscatter coefficient value from January 2015 to December 2019, and *UB*_*k*_ is the inverse value (from December 2019 to January 2015). When an intersection occurs between *UF*_*k*_ and *UB*_*k*,_ the value falls within the 95% confidence interval (*U*_*0.05*_ = ±1.96), and then the corresponding times of the intersection are considered the installation dates of the wind turbine foundations. This operation was carried out in MATLAB.

## Data Records

This dataset provides geocoded information about global OWTs from 2015–2019; it identified 6,924 wind turbines that comprise more than 10 nations. Data are available at 10 m spatial resolution, providing an explicit dataset for planning, monitoring and managing marine space. Global OWT dataset are publicly available for download from Figshare^22^ and can be visualized at https://arcg.is/0zu09X using an active ArcGIS online account.

The global OWT dataset is referenced to the WGS84 datum and stored in Shapefile (.shp) format. Each record consists of seven attributes: centroid latitude (centr_lat), centroid longitude (centr_lon), continent, country, sea area (sea_area), appearance year (occ_year) and month (occ_month). Description of these are tabulated in Table 1.

**Table 1:** List of attributes and their descriptions in the global OWT dataset.

No.	Attributes	Data type	Description
1	centr_lat	Float	Latitude of the wind turbine centroid
2	centr_lon	Float	Longitude of the wind turbine centroid
3	continent	String	Continent on which the wind turbine is located
4	country	String	Country in which wind turbines is located
5	sea_area	String	Sea area in which the wind turbine is located
6	occ_year	Integer	Year in which the wind turbine foundations were installed (yyyy)
7	occ_month	Integer	Month in which the wind turbine foundations were installed (mm)

## Technical Validation

### Method performance assessment

OWTs extraction is subject to uncertainties that arise from various background factors in the analysis grid, including tidal flats, turbidity of water bodies, and floating or temporarily mobile objects. Thus, to assess whether the extraction method has a high performance and whether the OWTs result outputted from GEE has a strong stability, we perform a sensitivity analysis of the wind turbine extraction against increasing SAR images to reveal that the amount of SAR image data that we utilized is enough to ensure the stability of the extracted results with various background factors. By calculating the precision (P) (Eq. (4)), which refers to the extracted real wind turbine number relative to the total extracted wind turbine number, recall (R) (Eq. (5)), which refers to the extracted real wind turbine number relative to the total real wind turbine number, and the comprehensive evaluation index (C) (Eq. (6)), which integrates the P and R value, we quantitatively evaluate the robustness of the extraction method.

**Fig. 5:**
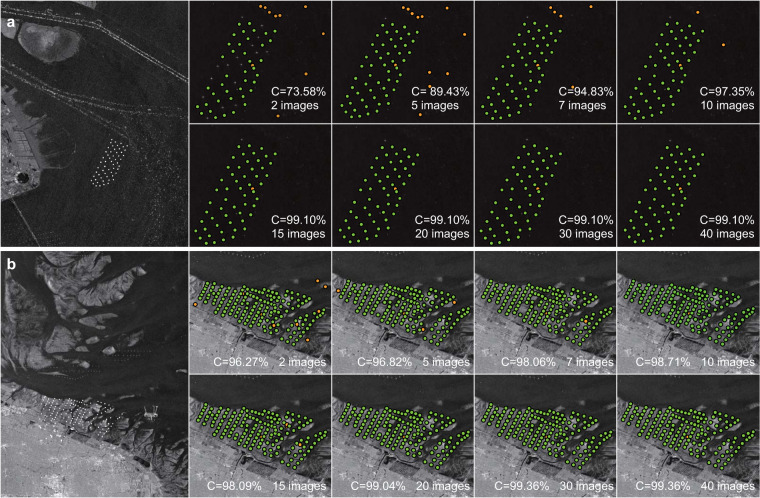
Sensitivity analysis of the comprehensive evaluation index on wind turbine extraction against increasing the SAR images at (**a**) Shanghai Lingang Demonstration Wind Farm and (**b**) Jiangsu Rudong Offshore Intertidal Demonstration Wind Farm, China. The wind turbine objects accurately identified by our method are represented by a solid green point, and the falsely identified wind turbine objects are represented by a solid orange point.

Using 1 to 40 images in the 2019 SAR image collection, Fig. 5 displays two examples of the extracted accuracy change for turbid water bodies and tidal flat backgrounds along the Shanghai coast (Shanghai Lingang Demonstration Wind Farm) and the Jiangsu coast (Jiangsu Rudong Offshore Intertidal Demonstration Wind Farm), China. The results reveal that the comprehensive evaluation index of the extracted wind turbines increased from 21.88% to 99.10% when 15 images were applied to the Shanghai Lingang Demonstration Wind Farm and increased from 83.78% to 99.04% when 20 images were applied to the Jiangsu Rudong Offshore Intertidal Demonstration Wind Farm. Since the Sentinel-1 satellite has a 12-day or 6-day revisit cycle, our analysis results indicate that using an annual average backscattering coefficient (covering more than 20 images) for OWTs extraction can ensure an extraction accuracy greater than 99% regardless of the background.

4 $${\rm{P}}=\frac{TP}{TP+FP}$$

5 $${\rm{R}}=\frac{TP}{TP+FN}$$

6 $${\rm{C}}=\frac{2\times P\times R}{P+R}$$

where TP is the number of accurately identified wind turbine objects, FP is the number of falsely identified wind turbine objects, and FN is the omission number of wind turbine objects.

### Accuracy assessment

Validation of the global dataset was conducted using an independent accuracy assessment approach. Here, we generated a validation set that consisted of 50 random offshore wind farms, covering 2,663 wind turbines using three methods. Reference data include (1) the high-resolution aerial imagery and Google images; (2) the comparison and corroboration across multiple source datasets, including the OWTs in the 4 C Offshore^17^, USGS USWTD^15^, UK REPD^16^, EMODnet^19^, OPSD^13^ and GBWSF^14^ databases; and (3) a comprehensive visual examination and an extensive internal review by the authors using Sentinel 2-MSI data or Landsat 8-OLI imagery with true colour composition and Sentinel 1 data after floating or temporarily mobile object removal.

**Fig. 6:**
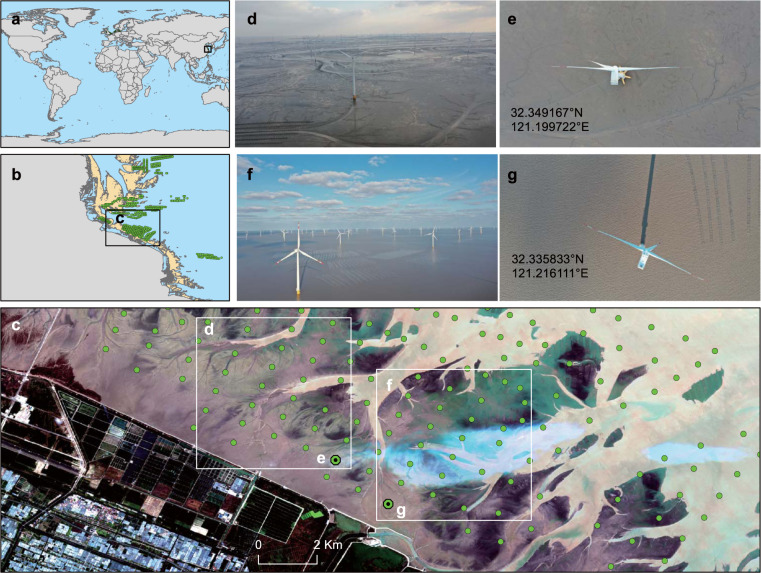
Jiangsu Rudong Offshore Intertidal Demonstration Wind Farm validation by unmanned aerial vehicle (UAV) aerial photography in 2019 along the coast of Jiangsu, China. (**a**–**c**) are the positions of this region in global areas, (**d** and **f**) are the side-view aerial photography images at the validation sites, and (**e** and **g**) are top views of the individual wind turbines in (**a** and **b**), respectively.

**Fig. 7:**
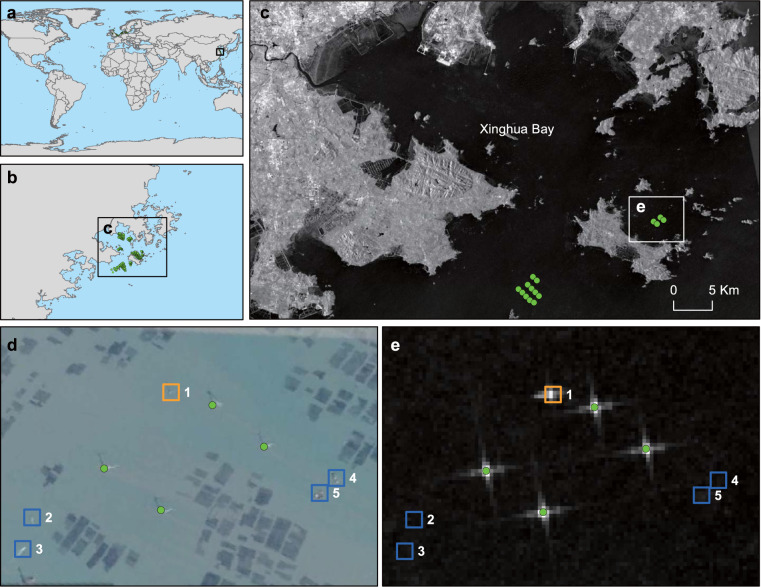
OWTs validation using 1 m high-spatial-resolution imagery from Google Maps obtained on 2016-12-12 at the Longyuan Putian Nanri island demonstration wind farm, Xinhua Bay, China. (**a**–**c**) are the positions of this region in global areas, the background map in d is Google high-resolution imagery, and the background map in e is 23 SAR image composited data after floating or temporarily mobile object removal. The wind turbines are represented by a solid green point, and the objects in blue boxes in **d** and **e** represent floating or temporarily mobile objects, including the ships. The objects in the orange boxes in d and e represent the offshore substation, which is easy to extract with wind turbines.

The use of aerial imagery for verification was conducted for October 2019. One offshore wind farm on the Jiangsu coast, China, covering 155 wind turbines, was validated by unmanned aerial vehicle (UAV) aerial photography images collected by a ground unmanned real-time kinematic (RTK) drone. All the photography images have geographic information, and Fig. 6 shows the specific location information of two wind turbines in that large wind farm. Furthermore, six other wind farms in China were also cross-validated with Sentinel 2-MSI data, Landsat 8-OLI imagery and Google high-resolution imagery in Google Earth (Fig. 7). In addition, 43 wind farms in North America and Europe covering six countries were selected, referenced and cross-validated using different national/international dataset sources. All the wind turbines (covering 50 wind farms) in the validation dataset were double examined for visual inspection using the Sentinel 1 data. Specifically, two authors who had sufficient backgrounds in remote sensing and GIS separately obtained these data source images country by country from the GEE platform and cross-validated the position and number of OWTs. The validation dataset is also publicly available for download from Figshare^22^.

The use of three methods to generate the independent validation dataset was motivated by the lack of a consistent set of global outvalidation data of OWTs for accuracy assessment. To report the precision metric, we calculated the ratio of accurately identified wind turbine objects to all detected objects in our dataset. The precision of the dataset is 100.00%, 99.54%, 99.09%, 99.71%, 99.48%, 99.62%, and 99.84% in the United States (number of wind farms = 1, number of wind turbines = 5), the United Kingdom (number of wind farms = 21, number of wind turbines = 1,120), Germany (number of wind farms = 8, number of wind turbines = 536), Denmark (number of wind farms = 8, number of wind turbines = 388), China (number of wind farms = 7, number of wind turbines = 410), Sweden (number of wind farms = 2, number of wind turbines = 64) and the Netherlands (number of wind farms = 3, number of wind turbines = 140), respectively (Online-only Table 1). The identification error is attributed to the met mast and offshore substation located inside or near the wind turbines, which are extracted with the wind farm, such as the OWFs in EnBW Baltic 2 and Arkona, Germany. To calculate recall, we subtracted falsely detected objects from all detected objects and divided them by all instances (using the data in the validation dataset). As expected, all recall values reach 100%, meaning that there are no omission wind turbines in the validated areas (Online-only Table 1).

**Fig. 8:**
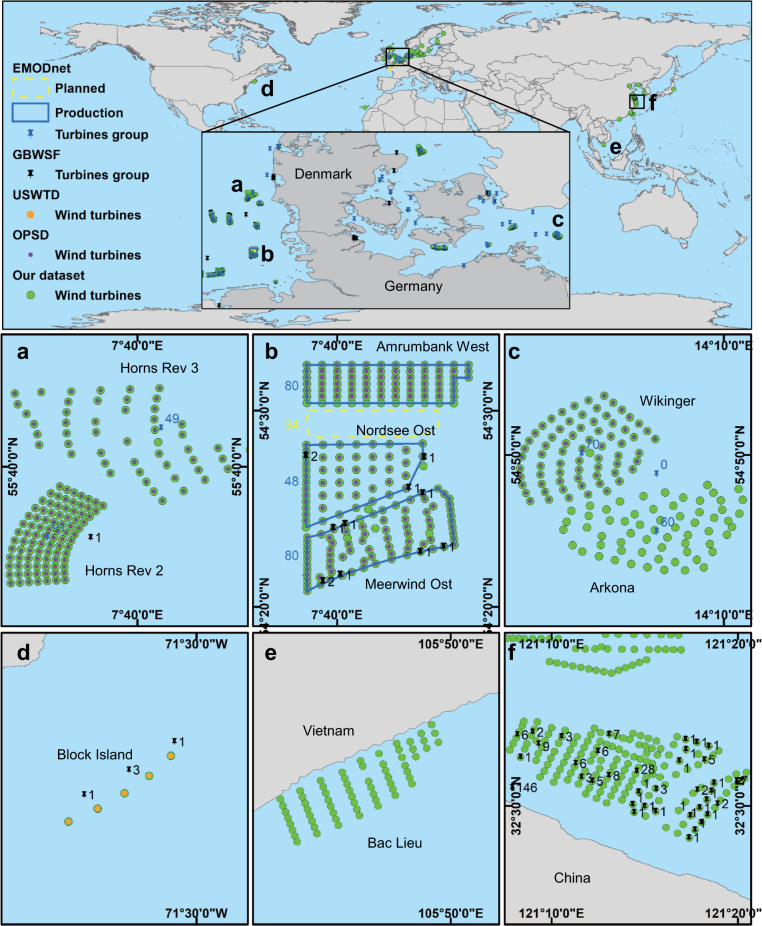
A visual wind turbine coverage and number comparison for our database with the GBWSF, USWTD, EMODnet, OPSD datasets in the wind farm in Germany, Denmark, the United States, Vietnam and China, including (**a**) Horns Rev 2 and 3, (**b**) Amrumbank West, Nordsee Ost, Meerwind Ost, (**c**) Wikinger and Arkona, (**d**) Block Island, (**e**) Bac Lieu, and (**f**) Jiangsu Rudong. The wind turbines in our dataset are represented by solid green points; the wind turbines in the OPSD dataset are represented by solid purple points; the wind turbines in the USWTD dataset are represented by solid orange points; and the wind turbines in the GBWSF dataset are represented by black pushpins. The wind farm status in the EMODnet dataset is shown by points and polygons with different colours. The yellow box indicates the plan status; the blue box indicates the production status; and the blue pushpins indicate the centroid of the wind farm. All are labelled with the corresponding wind turbine numbers.

Therefore, our validation shows that studies that use this OWT dataset need to note the purpose that these data serve. If the met mast and offshore substation near the wind farm do not matter, then this dataset has an acceptable accuracy. Compared to other databases that only provide approximate spatial location information and turbine numbers^16,19^ or incomplete, inaccurate information^14^, our dataset has a high resolution spatiotemporally. A visual comparison (Fig. 8) with the GIS OWTs data in the GBWSF, USWTD, EMODnet, and OPSD datasets can also confirm that our dataset has good coverage and high location accuracy and can further complement other databases as a consistent set of globally full coverage and high credibility OWT datasets.

## Usage Notes

The dataset derived from satellite imagery provides the spatiotemporal distribution of global OWTs from 2015 to 2019. This dataset has the potential to further elucidate the impact of OWTs on coastal ecosystems, support biodiversity conservation and environmental impact assessments, and help generate sustainable development strategies for offshore wind energy.

We take no responsibility for any third-party use or analysis of the data, nor do we endorse any third-party opinions or conclusions reached using these data. We also ask that users notify the authors of any errors or omissions identified in the data so that they can be corrected.

## Data Availability

All the code and processing scripts used to produce the results of this paper were written in GEE, MATLAB. Links to scripts and data for analyses can be found in the GitHub repository at https://github.com/tzhang-edu/GOWT.

## Online-only Table

**Online-only Table 1:** Different database comparisons (i.e., 4 C Offshore Wind Database, United States Geological Survey (USGS) Wind Turbine Dataset (USWTD), United Kingdom Renewable Energy Planning Database (UK REPD), Open Power System Data (OPSD) renewable power plant database, European Marine Observation and Data Network (EMODnet) wind farm database and the global datasets of wind and solar farm (GBWSF) built by Dunnett *et al*.) for 50 wind farms and the accuracy assessment of our dataset using the independent verification set in seven countries, including the United States, the United Kingdom, Germany, Denmark, China, Sweden, and the Netherlands.

Country	ID	Site name	Database comparison (wind turbine numbers)						Validation dataset	Accuracy assessment						
			4 C Offshore (Free access text data obtained online)	USGS USWTD (point data)	UK REPD (text data)	OPSD (point data)	EMODnet (point data or polygon data)	GBWSF by Dunnett *et al*. (point data)	TP	Our dataset (point data)	FP	FN	Precision (P)	Recall (R)	Comprehensive evaluation index (C)	Average
United States	1	Block Island	5	5				5	5	5	0	0	100.00%	100.00%	100.00%	100.00%
United Kingdom	2	Rhyl Flats	25		25	25	25	data missing	25	25	0	0	100.00%	100.00%	100.00%	99.54%
	3	Gwynt y Mor	160		160	160	160	data missing	160	160	0	0	100.00%	100.00%	100.00%	
	4	North Hoyle	30		30	30	30	data missing	30	30	0	0	100.00%	100.00%	100.00%	
	5	Burbo Bank Extension (Burbo Bank 2)	32		32	32	32	data missing	32	34	2	0	94.12%	100.00%	96.97%	
	6	Burbo Bank	25		25	25	25	data missing	25	25	0	0	100.00%	100.00%	100.00%	
	7	Barrow	30		30	30	30	30	30	31	1	0	96.77%	100.00%	98.36%	
	8	Robin Rigg West	58		28	28	30	1	30	30	0	0	100.00%	100.00%	100.00%	
	9	Robin Rigg East			30	30	30	2	29	29	0	0	100.00%	100.00%	100.00%	
	10	Hywind Scotland Pilot Park (Hywind 2) Demonstrator	5		5	data missing	5	data missing	5	5	0	0	100.00%	100.00%	100.00%	
	11	European Offshore Wind Deployment Centre (EOWDC) (Aberdeen Bay - Demonstration site)	11		11	data missing	11	data missing	11	11	0	0	100.00%	100.00%	100.00%	
	12	Teeside	27		27	27	27	data missing	27	27	0	0	100.00%	100.00%	100.00%	
	13	Inner Dowsing	27		27	27	27	data missing	27	27	0	0	100.00%	100.00%	100.00%	
	14	Lincs	75		75	75	75	data missing	75	76	1	0	98.68%	100.00%	99.34%	
	15	Lynn	27		27	27	27	data missing	27	27	0	0	100.00%	100.00%	100.00%	
	16	Sheringham Shoal	88		88	88	88	data missing	88	90	2	0	97.78%	100.00%	98.88%	
	17	Scroby Sands	30		30	30	30	data missing	30	30	0	0	100.00%	100.00%	100.00%	
	18	London Array	175		175	175	175	data missing	175	177	2	0	98.87%	100.00%	99.43%	
	19	Thanet	100		100	100	100	data missing	100	101	1	0	99.01%	100.00%	99.50%	
	20	Gunfleet Sands 1 and 2	48		48	48	48	data missing	48	48	0	0	100.00%	100.00%	100.00%	
	21	Rampion	116		116	116	116	data missing	116	117	1	0	99.15%	100.00%	99.57%	
	22	Ormonde	30		30	30	30	data missing	30	31	1	0	96.77%	100.00%	98.36%	
Germany	23	EnBW Baltic 1	21			21	21	data missing	21	21	0	0	100.00%	100.00%	100.00%	99.09%
	24	EnBW Baltic 2	80			80	80	data missing	80	82	2	0	97.56%	100.00%	98.77%	
	25	Arkona	60			60	60	data missing	60	62	2	0	96.77%	100.00%	98.36%	
	26	Amrumbank West	80			80	80	data missing	80	80	0	0	100.00%	100.00%	100.00%	
	27	Nordsee Ost	48			127	48	4	48	50	2	0	96.00%	100.00%	97.96%	
	28	Meerwind Ost	80				80	8	80	81	1	0	98.77%	100.00%	99.38%	
	29	Gode Wind 1 and 2	97			97	97	5	97	99	2	0	97.98%	100.00%	98.98%	
	30	Wikinger	70			70	70	data missing	70	71	1	0	98.59%	100.00%	99.29%	
Denmark	31	Nissum Bredning	4			4	4	4	4	4	0	0	100.00%	100.00%	100.00%	99.71%
	32	Rønland	8			8	8	9	8	8	0	0	100.00%	100.00%	100.00%	
	33	Anholt	111			111	111	data missing	111	112	1	0	99.11%	100.00%	99.55%	
	34	Avedøre Holme	3			3	3	data missing	3	3	0	0	100.00%	100.00%	100.00%	
	35	Middelgrunden	20			20	20	20	20	20	0	0	100.00%	100.00%	100.00%	
	36	Nysted	72			72	72	data missing	72	73	1	0	98.63%	100.00%	99.31%	
	37	Rodsand II	90			90	90	data missing	90	91	1	0	98.90%	100.00%	99.45%	
	38	Horns Rev 1	80			80	80	data missing	80	81	1	0	98.77%	100.00%	99.38%	
China	39	Shanghai Lingang	55					data missing	56	56	0	0	100.00%	100.00%	100.00%	99.84%
	40	Jiangsu Rudong	155					122	155	156	1	0	99.36%	100.00%	99.68%	
	41	Guodian Zhoushan Putuo District	63					data missing	63	64	1	0	98.44%	100.00%	99.21%	
	42	Zhuhai Guishan Hai	37					data missing	34	34	0	0	100.00%	100.00%	100.00%	
	43	Dalian Zhuanghe	73					data missing	73	73	0	0	100.00%	100.00%	100.00%	
	44	Longyuan Putian Nanri Island	4					data missing	4	4	0	0	100.00%	100.00%	100.00%	
	45	China-SPIC Binhai North H1	25					data missing	25	25	0	0	100.00%	100.00%	100.00%	
Sweden	46	Kårehamn	16			16	16	3	16	16	0	0	100.00%	100.00%	100.00%	99.48%
	47	Lillgrund	48			48	48	48	48	49	1	0	97.96%	100.00%	98.97%	
Netherlands	48	Prinses Amaliawindpark	60				60	data missing	61	61	0	0	100.00%	100.00%	100.00%	99.62%
	49	Egmond aan Zee	36				36	data missing	36	36	0	0	100.00%	100.00%	100.00%	
	50	Eneco Luchterduinen	43				43	data missing	43	44	1	0	97.73%	100.00%	98.85%	

TP is the number of accurately identified wind turbine objects, FP is the number of falsely identified wind turbine objects, and FN is the omission number of wind turbine objects.
